# Natural killer cell awakening: unleash cancer-immunity cycle against glioblastoma

**DOI:** 10.1038/s41419-022-05041-y

**Published:** 2022-07-08

**Authors:** Minjie Wang, Zijie Zhou, Xuan Wang, Chaocai Zhang, Xiaobing Jiang

**Affiliations:** 1 Department of Neurosurgery, Union Hospital, Tongji Medical College, Huazhong University of Science and Technology, Wuhan, 430022 China; 2 Department of Neurosurgery, Hainan General Hospital/Hainan Affiliated Hospital of Hainan Medical University, Haikou, 570311 China

**Keywords:** Cancer, Immunology

## Abstract

Due to the negligence of the complex tumor immune microenvironment, traditional treatment for glioblastoma has reached its limitation and cannot achieve a satisfying outcome in the past decade. The emergence of immunotherapy based on the theory of cancer-immunity cycle has brought a new dawn to glioblastoma patients. However, the results of most phase II and phase III clinical trials are not optimistic due to the simple focus on T cells activation rather than other immune cells involved in anti-tumor immunity. NK cells play a critical role in both innate and adaptive immunity, having the ability to coordinate immune response in inflammation, autoimmune disease and cancer. They are expected to cooperate with T cells to maximize the anti-tumor immune effect and have great potential in treating glioblastoma. Here, we describe the traditional treatment methods and current immunotherapy strategies for glioblastoma. Then, we list a microenvironment map and discuss the reasons for glioblastoma inhibitory immunity from multiple perspectives. More importantly, we focus on the advantages of NK cells as potential immune regulatory cells and the ways to maximize their anti-tumor immune effect. Finally, our outlook on the directions and potential applications of NK cell-based therapy combining with the advance technologies is presented. This review depicts NK cell awakening as the precondition to unleash the cancer-immunity cycle against glioblastoma and elaborate this idea from biology to clinical treatment.

## Facts

- Glioblastoma is one of the most aggressive and rapidly fatal tumor types in human brain.
- Traditional strategies have reached their limits in prolonging overall survival of glioblastoma patients.
- Current immunotherapies for glioblastoma did not show significant curative effect.
- NK cells activate the whole anti-cancer immunity and make up for single T cell activation.

## Open questions

- How are NK cells regulated in the glioblastoma microenvironment?
- What should be done to reprogram the dysfunctional NK cells infiltrated in glioblastoma?
- Lacking MHC I complex is one of the key features for NK cell activation and T cell inactivation, how can we achieve a balance for maximum anti-cancer immunity?

## Traditional strategies for glioblastoma

Maximal safe resection is the top priority among glioblastoma (GBM) therapeutic schedules, removing tumor-occupying effect and helping to identify the cancer histopathology and molecular pathology which are the basis for precise postoperative adjuvant treatment. However, high invasiveness and inevitable residue of GBM always escape from simple surgical resection, and patients without postoperative adjuvant therapy suffer from tumor recurrence after a short period of time. Hence, postoperative adjuvant therapies are used to target residual tumor cells and delay tumor recurrence. Temozolomide (TMZ) is an alkylating agent with antitumor activity and exerts cytotoxic effects through the mismatch repair of methylated DNA [1]. TMZ is the most commonly used chemotherapeutic drug due to its efficient blood-brain barrier penetration. As it antagonizes the repair enzyme O^6^-methylguanine-DNA methyltransferase (MGMT), TMZ works more effectively on patients whose tumors harbor a methylated MGMT promoter. Radiotherapy can produce ionizing radiation and damage the DNA of cancer cells. The standard postoperative radiotherapy regimen for GBM in adults is 60 Gy divided into 30 fractions after surgery. In elderly GBM patients, hypofractionation with a 45 Gy dose (more than 2 Gy per fraction) is also recommended. Concurrent use of TMZ with radiotherapy for at least six cycles is the standard postoperative adjuvant therapy for GBM patients. Additionally, targeted therapy, as a treatment modality from the cellular molecular level, has also been widely researched in recent years. Usually, it directly kills cancer cells by targeting specific genes and is mainly applied in clinical recurrent GBM patients. The most commonly used targeted drug is bevacizumab (Bev), which is the antagonist of VEGF-A and approved by the FDA [2]. However, most drug treatments only focus on tumor cells and ignore the whole abnormal tumor microenvironment (TME) network, including abnormal angiogenesis, hypoxic microenvironment, low pH, the existence of glioma stem cells, and more seriously, the relatively inhibitory immune microenvironment [3]. This is the key reason for the poor therapeutic effect. Therefore, taking tumor immunity as a breakthrough to reverse the whole inhibitory microenvironment network has great potential and numbers of immunotherapies appeared in the past decade.

## Current immunotherapies for glioblastoma

In 2013, Chen and Mellman firstly raised the concept of “cancer-immunity cycle”, revealing a series of stepwise immunity events that must be initiated for effective killing of cancer cells [4]. More recently, immunotherapy has been widely applied in cancers and achieved satisfactory results in hematological malignancies and several solid tumors. However, in addition to oncolytic viruses, the core of most immunotherapies, including immune checkpoint inhibitors, chimeric antigen receptors (CAR) T cells and vaccine therapy, mainly rely on the way of killing tumor cells by activating cytotoxic T cells. Unfortunately, all GBM immunotherapies have failed to provide persistent efficacy in phase II/III clinical trials. Thus, researchers conclude that although T cell activation plays an indispensable role in anti-tumor immune effect, it is far from reversing the whole adverse tumor microenvironment network, especially for GBM.

### Immune-checkpoint inhibitors

Drugs that reverse the activity of negative regulatory pathways limiting T cell activation are one of the most successful breakthroughs in malignancy treatment in the past decade. The common treatment targets include PD-L1 which are mostly expressed on a tumor cell, or PD-1 and CTLA-4 express on T cell. However, the overall therapeutic effect of immune-checkpoint blockade (ICB) in GBM is not optimistic, and the therapeutic effect varies greatly, which may be related to the relatively differential expression of PD-L1 in GBM and the individuals difference mutation load of tumor cells. More recently, results from a phase III clinical trial enrolling 369 patients with recurrent GBM showed that blocking PD-1 with nivolumab did not prolong median overall survival compared to bevacizumab (9.8 vs 10.0 months, *P* = 0.76) [5]. Similarly, pembrolizumab was proved ineffective as monotherapy for recurrent GBM in a recent phase II clinical trial enrolling 80 GBM patients [6]. In addition, many immunotherapeutic drugs are still in primary preclinical research stage. Therefore, more preclinical and clinical studies are needed to explore how to improve the efficacy of ICB in GBM.

### Vaccine therapy

Since GBM rarely metastasize outside the central nervous system, it is a potential cancer treatment strategy to activate the immune surveillance function of the brain through strengthening the adaptive arm of the immune system, especially for vaccines. Presently, it is mainly divided into peptide vaccine and dendritic cell (DC) vaccine. The common immunogenic targets of the former one including epidermal growth factor receptor (EGFR) vIII, isocitrate dehydrogenase (IDH)-1 (R321H), interleukin (IL)-13Rα2 and gp100. Some vaccination approaches have reached phase III clinical development, but the treatment effect is not satisfactory. Rindopepimut, a vaccine targeting EGFRvIII, did not increase survival than the control group in patients with newly diagnosed GBM (ndGBM) (20.1 vs 20 months, *P* = 0.93) according to the result of Phase III clinical trial enrolling 741 patients [7]. While in a latest phase III clinical trial enrolling 331 patients, an autologous tumor lysate-pulsed dendritic cell vaccine (DCVax®-L) combined with standard therapy extended mean overall survival to 23.1 months [8]. However, the clinical benefit of DC vaccines is not always obtained due to their unstable biological activity.

### Chimeric antigen receptor T cell therapy

Genetically modified T cells, which the CARs were engineered to express on T cell, it can be specifically recognized tumor cell surface antigen independent of major histocompatibility complex (MHC) exposure. What’s more, CAR-T cells can be edited to have an activated phenotype. These characteristics can help to overcome immunologic suppression to some extent. It is especially critical for GBM, a highly heterogeneous tumor with a complex tumor microenvironment. Presently, initial clinical experiences of CAR-T therapy for GBM have only three target antigens (EGFRvIII, HER2 and IL-13Rα2, which are short for epidermal growth factor receptor variant III, human epidermal growth factor receptor 2, and interleukin-13 receptor alpha 2 respectively) [9]. Their effects restrictively depend on the exclusive expression of antigen on cancer cells and vary a lot among several phases I clinical trials [10, 11]. Inhibitory immune compensation and antigen escape after CAR-T therapy may be two important reasons to hinder the efficacy of GBM treatment. By engineering multiple tumor-associated antigen recognition receptors on the surface of T cells or activating other anti-tumor immune cells in TME, it is expected to obtain better immunotherapeutic effect in the future.

### Oncolytic virus therapy

Oncolytic virus therapy can not only directly kill tumor cells, but also activate antitumor immune effect by destroying tumor cells and releasing tumor related antigens. In addition, viruses usually activate macrophages through Toll-like receptors and the activated macrophages cells can promote the infiltration of T cells into tumors [12]. However, as a rising method, the oncolytic virus has not achieved enough clinical experience. Results from a few phase I clinical trials demonstrated a remarkable effect for GBM patients, but researches about its safety and efficacy are under further investigation [13].

## A GME map involved in suppressed cancer-immunity cycle

Glioma tumor microenvironment (GME) is a complex and diversified network system, including tumor cells, immune cells, stromal cells, vascular endothelial cells and so on. In addition, with the advancement of tumor multi-omics research, the metabolic characteristics and tumor mechanism microenvironment of glioma have also attracted much attention. These are the main reasons leading to tumor immunosuppression and drug tolerance.

### Hypoxic microenvironment

The Nobel Prize in Physiology or Medicine in 2019 awarded the research of cellular sensation and adaptation to oxygen. Among them, hypoxia-inducible factor (HIF) plays a key role in orchestrating a metabolic switch that allows cells to survive in this environment. Hypoxia is a prominent feature in GBM due to the rapid growth of cancer cells and the unmatched angiogenesis and oxygen supply. Hypoxia always contributes to the malignant phenotype of GBM by reprogramming the cancer cells themselves and the tumor microenvironment [14]. For glioma cells, hypoxia is related to rapid proliferation, anti-apoptosis, invasion, resistance to chemoradiotherapy [15]. In GME, immature DCs can express many chemokine receptors and be expulsed from the tumor upon hypoxia [16]. Meanwhile, hypoxia downregulates MHC-II expression on DCs and abolishes the ability of DCs to activate T cells [17]. Further, hypoxia promotes the infiltration of high levels of immunosuppressive cells, such as tumor associated macrophages (TAMs), myeloid-derived suppressor cells (MDSCs), and regulatory T cells (Tregs) [18–20]. Several immune checkpoint proteins also increase in a HIF-1α dependent manner, which causes T cell exhaustion in cancer [21, 22]. Recently, Lin et.al established a hypoxia risk signature consisting of VEGFA, HK2, JUN, LDHA and GAPDH [23]. This signature showed strong power for prognosis assessment and patients with low hypoxia risk scores survived longer, genes involved in the negative regulation of the cancer-immunity cycle were mostly upregulated in the high hypoxia risk group. Hence, correcting the hypoxia in GBM is an important prerequisite for persistent immunotherapy efficacy.

### Suppressive immune microenvironment

GBM is infiltrated with amounts of suppressive immune cells such as microglia, macrophages, MDSCs and Tregs, and the immune cell composition differs based on the IDH mutation status, the telomerase reverse transcriptase (TERT) promoter mutation and transcriptional subtypes [24]. The suppressive immune microenvironment helps GBM avoid immune destruction and promotes initiation and recurrence. Reprogramming GBM immune microenvironment from “cold” to “hot” is critical for the persistent response to immunotherapy. Fu et al. revealed the complexity of GBM immune microenvironment with single cell sequencing technology [25]. The results demonstrated that mononuclear phagocytes and T lymphocytes took up the majority of immune cells in GBM, with the former ones significantly increased and the latter significantly decreased when compared with that in peripheral blood mononuclear cells (PBMCs). Microglia and macrophages infiltrated the most in GBM, they were divided into 13 subgroups based on surface markers and highly heterogeneous depending on differential expressions of immune checkpoints, immunosuppressive cytokines and other tumor-promoting factors (TGF-β, IL-10, VEGF et al.). Unexpectedly, traditional M1-like macrophage markers also co-expressed pro-tumor markers such as IDO and PD-L1, which reflected that macroglia and macrophages in GBM may not be simply defined with previous dichotomy. T cells in GBM also demonstrated diversity according to the surface markers, exhausted subsets (PD-1, LAG-3 and TIM-3 positive) and Tregs increased in GBM. NK cells infiltrated in GBM more smoothly than T cells, but their cytolytic ability were severely disturbed, which enlightened that promoting NK activity could be a potential anti-GBM method. Insights into distinct GBM immune microenvironments partially contribute to immunotherapy, but more related clinical trials are still required.

### Metabolism microenvironment

Aberrant metabolisms of glucose, lipid and amino acid are hallmarks of GBM, accompanying with the accumulation of amounts of metabolites. Lactate, reactive oxygen species (ROS) and lipid are three focused metabolites which affect malignant and normal cells to a great extent. Lactate metabolism depends on aerobic glycolysis (Warburg’s effect), indicating that cancer cells produce lactate after absorbing glucose as a substrate for oxidative phosphorylation, even under normoxia [26]. The lactate produced by cancer cells is further secreted into the extracellular space and functions in cancer progression by creating an acidic niche. Extracellular acidosis also suppresses T cell cytotoxicity, reduces the expression of IL-6, CCL2 and TNF-α in M1-like macrophages, but increases the expression of CD163, CD206 and Arg-1 in M2-like macrophages, other anti-cancer immune cells like NK and DC cells dysfunction likewise after sensing high level lactate in TME [27]. All cells in local TME can generate ROS, and the increased ROS can reversely inhibit the cancer cell proliferation. However, as cancer cells evolve, they develop the resistance and even addiction to ROS, leading to immune escape [28, 29]. ROS yields post-translational modification of cysteine residues in proteins, which may alter antigenicity and cause T cell escape. Meanwhile, high level ROS promotes the immune checkpoints expression on cancer cells to inactivate NK and T cells [29]. Further, the immune cells in TME are totally intolerant to ROS and presented with many dysfunctions, such as lymphocytes anergy and apoptosis, reduced antigen presentation, increased Tregs and MDSCs infiltration [30]. Lipids, as basic energy reservoirs, also play key roles in fundamental cell biological processes. In cancer, lipids have direct impacts on immune cells apart from malignant cells [31]. High lipid contents in DC block the expression of MHC class I-peptide complex and inhibit tumor antigen cross-presentation [32]. Membrane cholesterol efflux in TAM activate STAT6 and PI3K signaling to enhance IL-4 mediated pro-tumor function and positively relate to CD8+ T cell exhaustion [33, 34]. Reprogramming metabolism in TME may be a potential target for sensitizing immunotherapy in GBM.

### Mechanical microenvironment

Considering that GBMs are a lot stiffer than normal brain tissues, mechanical microenvironment is a specific property and becomes novel research target derived from the accumulation of extracellular matrix. Mechanical microenvironment contains physical components such as collagen and fibrin, and many intracellular signaling like integrin signaling. Mechanical microenvironment regulates the expressions of oncogenes and tumor suppressors, carcinogenesis, cancer progression and therapy resistances. Moreover, the immune system is found to promote the formation of mechanical microenvironment, especially the macrophages (CD68 and CD163 positive) infiltrated in cancer can secrete TGF-β, which mediates the production of collagen and activates LOX, leading to collagen crosslinking [35]. Recent researches also demonstrated that mechanical microenvironment could take effects on immune cells and impair their functions. Angel et al. proved that innate immune cells utilized PIEZO1 to respond to force and alterations in cyclical hydrostatic pressure [36]. Zhu et al. put forward that mechanical force can be applied on immunoreceptors to induce biophysical and biological effects on immune cells through changing ligand bonding, conformation and downstream signal transduction [37]. For example, mechanical force can change the conformation of bonds between T cell antigen receptor (TCR) and complexes of peptide and MHC, impairing T cells cytotoxic effect [38]. In addition, mechanical force also has key roles in prolonging the duration of antigenic interactions and integrating comprehensive signals for T cell activation. Nadia also demonstrated that the tension of cancer maintains their fast metabolism, contributing to the heterogeneous specialized TME [39]. Hence, we must put the mechanical microenvironment into consideration when designing a novel immunotherapy.

## NK cell emerges as an innate modulator of cancer-immunity cycle

**Fig. 1:**
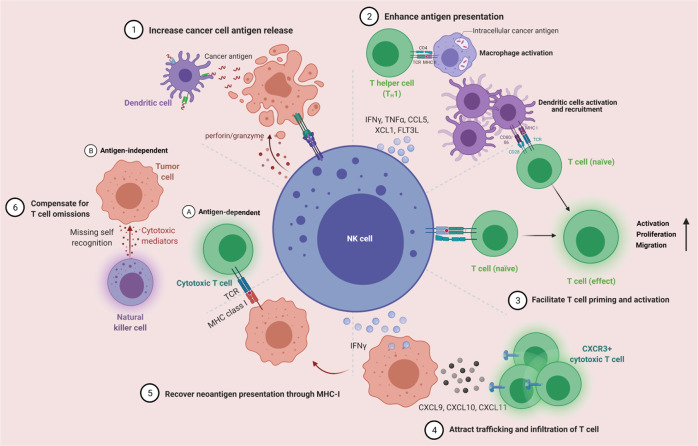
NK cells are activated to impacting almost every step of cancer-immunity cycle. Activated NK cells increase cancer cell antigen release, enhance antigen presentation, facilitate T cell priming and activation, attract T cell trafficking and infiltration, recover neoantigen presentation and compensate for T cell omissions.

Cancer-immunity cycle firstly presents the progress of cancer elimination by immune system, containing details from antigen recognition to cancer cell killing. In the past decade, most immunotherapies directly aimed at homogeneously activating T cell-based adaptive immunity to eliminate cancer cells, while several recent strategies focus on maximum activation of the innate immune cells because they are frontline responders of cancer cells and capable of rapidly mediating a response [40]. However, the efficiencies of activating innate immune cells are highly dependent on the infiltration of functional T cells in cancer, which are higher in tumors with high mutational loads and less in those with low ones such as GBM [41]. Exceptionally, NK cells not only can swiftly kill aberrant cells independent of T cells if they show surface markers associated with oncogenic transformation, but also enhance T cell responses, which support the role for NK cell as an all-powerful anticancer role. Specifically, while T cells only have a single T cell antigen receptor (TCR) and need to be activated by peptide-major histocompatibility complexes presented by antigen presentation cells (APCs) to trigger a suite of activating signaling cascade, NK cells have a wider range of receptors to recognize tumor cells which lack MHC/ human leukocyte antigen (HLA) expression. Meanwhile, NK cells are activated to generate comprehensive anti-cancer effect, impacting almost every step of cancer-immunity cycle (Fig. 1). When NK cells encounter tumor cells and are activated, they form a synapse with tumor cell and lytic granules converge towards the synapse through microtubules. Granules contain two cytotoxic effectors (perforin and granzyme) are released at the synapse. Perforin inserts itself into tumor cells and forms pores, leading to osmotic lysis, and granzyme transfers through the pores and activates caspases, causing apoptosis of tumor cells. These increase the release of cancer cell antigens and make way for APCs. In the stepwise part of cancer antigen presentation, NK cell-derived IFN-γ and TNF-α are proved to promote the activation and maturation of DCs and macrophages, which enhance the strength of antigen presentation [42]. Meanwhile, secretion of CCL5, XCL1 and FLT3L by NK cell attracts DCs to the tumor microenvironment, demonstrating the NK-DC axis in enhanced T cell response [43, 44]. For T cell priming and activation, Fan et al. proved that activated NK cells could facilitate the priming of tumor-specific CD8^+^ T cells in an IFN-γ–dependent manner, inducing their activation and proliferation [45]. Strikingly, Erokhina et al. revealed that HLA-DR^+^ NK cell was even able to directly take in and present certain antigens to CD8^+^ T cells as an APC [46]. After inhibiting PD-L1 on NK cell, it can boost spontaneous cross-priming of tumor Ag-specific CD8^+^ T cells by promoting DC activation [47]. Additionally, IFN-γ from NK cell stimulates cancer cells and induces their expression of CXCL9, CXCL10, and CXCL11. These ligands are all for CXCR3 and can contribute to downstream activation of LFA-1 and newly expressed integrins, resulting in increased trafficking and infiltration of CXCR3^+^ CTL [48]. Recently, Leah et al. showed that functional T cells were also recruited to tumors in an NK cell-dependent manner, in which process NK cell-derived CCL5 plays a key role [49]. In the recognition and killing part of cancer-immunity cycle, NK cells- derived IFN-γ promotes tumor neoantigen presentation through MHC-I to elicit anti-tumor T cell immunity, and modulates T cell activity directly. Furthermore, NK cells kill cancer cells independent of the expression of MHC molecules, producing a perfect complementary effect on T cell cytotoxicity. Above all, cancer-immunity cycle is strengthened by NK cells to initiate and orchestrate the activation of multiple immune cell subsets for productive antitumor immune responses [50].

## Strategies to augment NK cell activity

NK cell is considered as the bridge between innate immune system and adaptive immune system, and it seems to play a promising role in next generation immunotherapy for GBM. Hence, emerging researches which focus on modulating NK cell function to improve anticancer immune effect have been promoted. A number of clinical rials of NK-cell based immunotherapies in glioma are currently active in Table 1.

**Table 1:** A summary of clinical trials on NK cell-based immunotherapies for glioma.

Agent	Cell source	Treatment approach	Malignancy	Study phase (status)	Identifier (Trial name)
***CAR NK cells***					
Anti-MUC1 CAR-pNK cells	Autologous NK cells	Infusion	MUC1 + glioma	I/II (unknown)	NCT02839954
NK-92/5.28.z cells	NK-92 cells	Intracranial injection upon repeat surgery or biopsy	Recurrent or refractory HER2^+^ GBM	I (recruiting)	NCT03383978 (CAR2BRAIN)
***Non-engineered NK cells amplified in vitro***					
Autologous Natural Killer	Autologous NK cells	Infusion	Glioma	I (suspended)	NCT00909558
Autologous NK cells	Autologous NK cells	Intra-tumoral injection	Malignant glioma	I (not yet recruiting)	NCT04254419 (NK HGG)
Autologous expanded NK cells	Autologous NK cells	Infused intravenously into the ventricle	Recurrent childhood ependymoma	I (completed)	NCT02271711
Activated autologous NK cells in combination with rhIL-15	Autologous NK cells	Infusion	Advanced solid tumors of children and young Adults	I (completed)	NCT01875601
Donor NK Cell in combination with HLA-haploidentical HCT	NK cells from donors	Infusion	High grade glioma	II (active, not recruiting)	NCT02100891 (STIR)
***Lymphokine-activated killer (LAK) cells***					
Lymphokine-activated killer cells	Autologous PBMCs	Intra-tumoral injection	Primary GBM	II (withdrawn)	NCT00814593
Lymphokine-activated killer cells in combination with aldesleukin	Autologous PBMCs	I Intra-tumoral injection	Primary, recurrent, or refractory malignant gliomas	II (suspended)	NCT00003067
Aldesleukin-stimulated LAK cells	Autologous PBMCs	Intra-tumoral injection	Primary GBM	II (completed)	NCT00331526
Lymphokine-activated killer cells in combination with bispecific antibody MDX447	Autologous PBMCs	NA	GBM with EGFR expression on tumor cell surface	I (completed)	NCT00005813

Abbreviations in Table 1:

*CAR-pNK cells* CAR-peripheral NK cells, *GBM* glioblastoma, *rhIL-15* recombinant human interleukin-15, *HLA* human leukocyte antigen, *HCT* hematopoietic cell transplantation, *PBMCs* peripheral blood mononuclear cells *EGFR* epidermal growth factor receptor.

### Cytokines

Cytokines are crucial for NK cell differentiation, activation and immunological effect persistence, with their receptors expressed variously on NK cells depending on different states and functions. In GBM, immunosuppressive cytokines like TGF-β and IL-10, are dominant in tumor immune microenvironment (TIME), which inhibit NK cells directly or indirectly by inducing other immunosuppressive factors. Hence, a cluster of stimulatory cytokines are required to restore the attenuated NK cell function independently or cooperatively. IL-2 and IL-15 are the most widely studied cytokines in NK cells homeostasis and have many positive functional effects that enhance the antitumor response. IL-2 stimulate NK cells and CD8+ T cells via a dimeric IL-2R formed by IL-2Rβ (CD122) and IL-2Rγ (CD132) subunits, enhancing cell survival, proliferation, cytokine production and cytotoxic efficiency. HSP70/IL-2 treated NK cells cross the blood brain barrier and target tumor cells more effectively than untreated ones [51]. Although some researches show that IL-2 expands and activates immunosuppressive Tregs through higher affinity receptors IL-2Rα (CD25), recombinant mutant IL-2 is being put forward with decreased CD25 binding and increased CD122 binding capacity [52]. Meanwhile, strategies depleting Treg cells are also combined with IL-2 therapy [53]. IL-15 is another member of γ chain (γC) cytokines, it can stimulate proliferation and maintenance of NK cells like IL-2, increasing CD69 expression and IFN-γ, perforin, and granzyme B production. Moreover, IL-15 doesn’t induce Treg cell activity and has less toxicity, which means IL-15 are more compatible than IL-2 in immunomodulating. Additionally, several other cytokines in interleukin family like IL-21, IL-12, IL-18, IL-23, IL-27, IL-35 can also increase NK cell cytotoxicity, proliferation or synergize other cytokines for better expansion and activation [54]. Obviously, the modulation of NK cells through the cytokine treatment is a promising clinical direction, while the specific usage for GBM patients should be further studied according to the individual TIME situation.

### Inhibitory receptor blockade

NK cell activation is finely regulated by inhibitory receptors to avoid unnecessary or excessive immune responses. In cancer, these inhibitory receptors are often utilized to suppress NK cells function by cancer cells, leading to immune evasion [55]. HLA-class I-specific inhibitory receptors of NK cell assist discriminate healthy cells from tumor cells, which frequently decrease their expression of HLA-class I to restrain the tumor antigen presentation pathway. Cancers with low HLA-class I are more susceptible to NK cell elimination via the “missing-self” recognition, and blocking these receptors on NK cells would trigger their activity, leading to the rejection of the target cancer cells. Killer cell immunoglobulin-like receptors (KIRs), leukocyte immunoglobin-like receptors (LIRs), and natural killer group 2 A (NKG2A). These inhibitory receptors carry an inhibitory motif in their cytoplasmic domain called the immunoreceptor tyrosine-based inhibition motif (ITIM). Additionally, some other non-HLA-specific receptors also exist, such as PD-1, LAIR-1 and so on. Tumors activate these non-HLA-specific receptors through cell surface or extracellular matrix ligands, dampening the anti-tumor responses of NK cells. PD-1 expression was initially discovered on T cells, and on NK cells later [56]. PD-L1 and PD-L2 are two PD-1 ligands which are mainly expressed on tumor cells. Blocking PD-1 receptor with monoclonal antibody reactivates the function of T and NK cells in tumor and has achieved great clinical progress in several types of cancer. LAIR1 is a collagen binding inhibitory receptor expressed on immune cells [57, 58]. Tumor cells and associated stromal cells have elevated level of collagen, which may inhibit NK cytotoxicity and leads to immunosuppression. Most recently, NGM438, a novel antagonist antibody inhibiting LAIR1, is studied for the treatment of advanced solid tumors. In glioblastoma, CD155 has also been established as an immunomodulatory receptor, able to both activate NK cells through interactions with CD226 (DNAM-1) and CD96 and inhibit them through interaction with TIGIT [59]. Inhibiting glioma-derived galectin-1can stimulate endogenous antitumor NK cell activity in the absence of adaptive immunity [60]. Theoretically, any inhibitory receptor blockade mentioned above could unleash the NK cell anti-tumor immune response. Also, decreasing inhibitory receptors through genetic engineering has the same rationale. For example, knocking out TIM3 in NK cells enhances growth inhibitory effects on human glioma cells [61].

### CAR-NK cell therapy and multi-specific engager

As CAR-T cell therapy being proved effective in hematological malignancies and some solid tumors, CAR-NK therapy emerged in recent years with potential advantages over CAR-T therapy, such as less CAR antigen loss induced immune escape, higher human safety and the possibility to be an off-the-shelf allogeneic therapeutic [62]. Engineering NK cells to express CAR equips them with specific targeting and killing capacity. EGFRvIII and HER2 are two reported CAR antigens which can both exert marked effects in GBM [63, 64]. Recently, dual-specific CAR NK cells with several functional moieties targeting antigen escape, immunometabolic reprogramming of immune responses, and poor immune cell homing, are engineered to address the resistance of GBM to therapy [65]. Moreover, off-the-Shelf EGFR-CAR NK Cells have synergistic therapeutic effect on GBM combined with oncolytic virus [66]. Primary NK cells (from peripheral blood or umbilical cord blood), NK-92 cell line and induced pluripotent stem cells (iPSCs) are several main resources of NK cells. CARs guide NK cells homing and promote adhesion to targets, then CAR-NK can be activated once they interact with cancer cells expressing corresponding specific antigen. Recent research also unexpectedly proved that CAR-NK, rather than NK cell, could eliminate MDSCs and increase the efficiency of CAR-T therapy in solid cancer [67]. Multi-specific Engager technology is a targeted immuno-oncology platform that connects patients’ own NK cells to malignant cells. It is usually a small construct (50–75 kDa) and creates an efficient immunological synapse between NK cells and tumor cells [68]. Generally, bi- and tri-specific engagers that engage NK cell-activating receptors such as CD16, NKp30, NKp46, boost NK cell cytotoxicity against specific tumor targets [69]. Due to their increased biodistribution for their smaller size, lower immunogenicity and greater flexibility, multi-specific engagers have been applicated in some hematologic and solid tumors, providing foundation for the application in GBM [70–73].

### Memory-like NK cell and NK cell vaccine

It is generally accepted that innate immune responses of NK cells would not be amplified when exposed to the same target. However, this dogma has been challenged in the past decade, increasing evidence proved that NK cells could acquire “memory-like (ML)” characteristic of elevated immune response upon a secondary stimuli [74]. Generally, ML NK cells can be induced by specific receptors or cytokines, and have greater survival and proliferative potential. Hence, NK cell memory generation come with opportunities to translate NK cell recall responses into the clinic as cancer immunotherapy. Previously, NK cell immune memory was revealed to regulate adaptive immunity and explored to be harnessed by vaccination [75]. Nowadays, based on the principle that ML NK cells can increase the secretion of IFN-γ and TNF, recent preclinical study in leukemia has proved the feasibility of cell adoptive immunotherapy approaches with ML NK cells [76]. NK cell memory increases the IL-2Rα (CD25) expression, which provides fundamental basis for using low dose rhIL-2 as a supporting cytokine after adoptive therapy in clinical trials [77]. Compared to conventional NK cells, ML NK cells also have enhanced FcγRIIIa (CD16)-induced killing effects and attenuated inhibitory KIR signals, methods such as CD16A bi-specific engagers, NKG2A inhibitor and CAR engineering have achieved satisfied outcomes in Hodgkin lymphoma and leukemia [78–80]. Meanwhile, ML NK cells maintain their killing activity even in an immunosuppressive tumor microenvironment, which may have great prospects in solid tumors especially for GBM [81]. Nowadays, there are also several limitations in the application of ML NK cells. ML NK cells could be successfully obtained from human NK cells after a brief (12–18 h) preactivation with the IL-12, IL-15 and IL-18 cytokine cocktail, but maintaining them long-term in vitro without high-doses of cytokines is a technical challenge, which makes it hard to assess their potential in exhibiting memory because they cannot revert to a resting state in vitro [82]. On the other hand, when activated by cytokines, NK cells engage a robust metabolic response involving the upregulation of both glycolysis and oxidative phosphorylation (OXPHOS). Dysfunctional NK cell responses in disease states have been linked to failures in cellular metabolism. For instances, nutrient deprivation for NK cells in solid tumors can limit mTORC1 activity resulting in reduced glucose uptake and glycolysis in activated NK cells that would ultimately disrupt pro-inflammatory NK cell functions. Due to this, immunotherapies based on memory-like NK cells may not achieve satisfactory effects in some particular groups which were in metabolic disorders [83, 84]. Hence, overcoming these restrictions may maximize the effects of memory-like NK cells in future.

### Metabolism regulation

Repressed functional states of NK cells infiltrated in cancer have been studied to reprogram their activities impacted by TIME. Recent researches reveal that metabolic change is the key factor in immune cells fate, which affect their contribution in cancer progression [85–87]. Tumor-derived metabolites like adenosine and lactate, glucose restriction, amino acid depletion and hypoxia are main factors influencing NK Cell metabolism [88]. The latest result demonstrated that oxidative stress and glucose metabolic impairment drive NK cell dysfunction in tumors to a great extent [89]. Considering that transcription factor NRF2 is a master regulator of the cellular antioxidant response, activating the NRF2 antioxidant pathway can normalize NK cell glucose metabolism. Meanwhile, elevating STAT3 signaling can drive Warburg metabolic reprogramming in NK cell similar to tumor cells, and these metabolic adaptations would make them better tolerant of the TIME. Another research proved that lipid acids in TIME could be absorbed by infiltrated NK cells, which caused adaptive lipid metabolism increasing and impaired NK cell function [90]. Hence, inhibiting the lipid acids absorption and metabolism in NK cells may rescue their function in killing cancer cells. Recent study showed that TGF-β induced metabolic dysfunction of circulating NK cells and blocking TGF-β could restore NK cell metabolism and function [91]. In conclusion, cell metabolism is closely related to tumor immunity. Metabolic reprogramming is expected to more effectively activate the antitumor effect of NK cells.

### Others

In addition to the above ways, novel immunomodulatory drugs have been paid much attention in cancer immunotherapy and several kinds of drugs have been put forward to activate NK cells, such as small molecular inhibitors & activators and Chinese herbal medicines (CHMs). Deletion of Rb (a tumor suppressor) and overexpression of mutated Ras (a proto-oncogene) are enough to confer resistance to NK cell-mediated cytotoxicity in glioma cells, Rb activator or Ras inhibitor may reprogram the immune evasion [92]. Pharmacological inhibition of lysine-specific demethylase 1 (LSD1) increases NK cell killing in diffuse midline gliomas [93]. Cilengitide is the only integrin receptor antagonist which entered into the phase III clinical trial of glioblastoma treatment. It targets the integrin αvβ3 and αvβ5 receptors to inhibit angiogenesis and promote tumor apoptosis. It worked well in the preclinical study and phase I/II clinical trials of glioblastoma, but phase III clinical trials ended in failure. Recently, Cilengitide is innovatively used to sensitize glioblastoma stem cells to be lysed by NK cells [94]. Lenalidomide and pomalidomide are effective drugs for refractory myeloma and have been approved for clinical use by FDA in 2013. Their anti-cancer mechanisms are extensive and the newest one showed their specific function in triggering activation of Zap-70 in NK cells [95]. Meanwhile in glioma, N6-isopentenyladenosine (iPA), an isoprenoid modified adenosine with a well-established anticancer activity, was able to induce a significant upregulation of cell surface expression of NKG2D ligands on glioma cells, leading to enhanced NK cell degranulation [96]. Moreover, a latest systematic review evaluated the immunomodulation of CHMs and several CHMs are related to enhancement of NK cells activity such as *Panax ginseng, Angelica sinensis, Radix Astragali, Ganoderma lucidum* and *Lentinus edodes* [97]. In future, more drugs with various targets and immunomodulatory effects would be invented and applicated in caner due to their relative convenient local or systemic administration.

## Frontier technologies and potential applications in NK cell-based immunotherapy

### Current advanced glioma immune research model

Animal model has become a wildly used tool for immunotherapy efficiency validation in preclinical researches. Xenograft mouse model is one of the classic models in which human derived GBM cell line is engrafted in the immune-compromised mice. However, this model is not suitable for the study of T cells or NK cells due to deficiency of tumor microenvironment. While immune-competent mouse model can solve the problem of TME deficiency, but the injected mouse derived tumor cell lines cannot accurately reflect the real situation of human tumors. In patient-derived xenograft (PDX) model, tumor tissues which contain tumor cells, immune cells, stromal cells and vascular endothelial cells from patients are injected into nude mice, it not only solves the problem of lacking TME, but also reflects cancer individual features to a great extent. But this model is relatively time and financial consuming. Organoids are ex vivo three-dimensional (3D) models that are derived from human embryonic stem cells or patients derived tumor tissues, which have the ability to self-organize and can more realistic reflect both the structure and function of primary human organs [98]. More recently, Jacob et.al developed a rapid and reliable method to generate patient-derived GBOs, which precisely recapitulate the histology, gene mutation and heterogeneity of their corresponding parental tumors [99]. In addition, 3D bioprinting models are also promising 3D ex vivo tumor immune research models. Compared with organoids, they can effectively solve the problems of lacking blood vessels and single cell type [68,69]. Therefore, both organoid and 3D bioprinting may be a more effective preclinical model to evaluate the clinical efficacy and toxicity of NK cell-based therapy.

### ScRNA-seq and DSP

Due to their natural feature of scattered distribution, immune cells are more suitable for single-cell genomics, which present a potential to open up the way we explore immune cell heterogeneity and study their spatial organization, dynamics, clonal distribution, pathways, function and cross-talks [100]. For example, Crinier et al. utilized single-cell RNA sequencing (scRNA-seq) to reveal organ specific signature and heterogeneity of NK cells in the blood and spleen [101]. Ni et al. defined the transcriptional landscape of cancer-infiltrating NK cells with scRNA-seq and discovered that HIF-1α-deficiency maintained NK cells anti-tumor activity [102]. What’s more, new technology can solve the problem of lacking spatial information in scRNA-seq. Digital spatial profiling (DSP) is an integrated system comprising hardware, software and chemistry that enables simultaneous, highly multiplex spatial profiling of proteins or RNA in formalin-fixed, paraffin-embedded (FFPE) samples [103]. DSP uses next-generation sequencing (NGS) to achieve protein and RNA information in the region of interest (ROI) which covers 1 to ~5,000 cells. Combing scRNA-seq and DSP can help researchers understand the heterogeneity of solid tumors as well as immunocytes more comprehensive and precisely. Moreover, this may also have the potential to be used for diagnosis, prognosis and clinical decision.

### CRISPR-Cas9

The clustered regularly interspaced short palindromic repeat (CRISPR)-associated protein 9 (CRISPR-Cas9) system was firstly discovered in prokaryotes to resist against viruses [104]. Later, it was designed as a tool of gene editing and widely applied in the area of cancer immunotherapy. Two scientists also won the 2020 Nobel Prize in Chemistry for their significant contributions to CRISPR-Cas9. Freeman et al. performed genome-wide CRISPR/Cas9 screens and identified that IFN-γ signaling and antigen presentation limited NK cell anti-tumor activity [105]. Pomeroy et al. developed a highly efficient method for editing the genome of NK cells with CRISPR-Cas9 to knock out inhibitory signaling molecules such as ADAM17 and PDCD1 [106]. Recently, genetic reprogramming with CRISPR-Cas9 has been extended for NK cell cancer immunotherapy [107]. By virtue of its outstanding capability in gene-editing, CRISPR-Cas9 could knock out specific inhibitory receptor on NK cells to enhance their activities, knock in pan-specific CAR-like molecules for improved tumor recognition and tumor-specific chemokine receptor for NK cell infiltration. However, the mechanisms of NK cell dysfunction in tumor are not fully understood yet, and the in vivo CRISPR-Cas9 delivery is still challenging. In future, tumor microenvironment targeted therapy and novel drug delivery carriers should be exploited to endow CRISPR-Cas9 greater therapeutic potential.

## Future perspectives

Current accomplishments have fueled interest in improving anti-tumor immunity for cancer therapy. However, these therapies focused on enhancing the T-cell immune response did not produce the same anti-tumor effect in GBM. In addition, limited by the specific antigen recognition process of T cells and the high heterogeneity of glioma cells, GBM patients with ideal response to immunotherapy at first almost end up with immune evasion due to specific antigen loss. Therefore, it is urgent to explore new core immune cells and create more effective and durable next generation immunotherapy regiments. NK cells are the most potent innate immune cells able to activate adaptive immunity and can mobilize a variety of immune cells to exert anti-tumor effect. Their complementary advantages with T cells are expected to achieve a breakthrough in GBM immunotherapy. Even though NK cells are sometimes found to be dysfunctional or tolerogenic in the TME, improved knowledge of how NK cells are regulated in this context may allow therapeutic exploitation in the near future. Overall, NK cells are promising in the field of GBM treatment. Reasonable combination with various new technologies and equipment and learning more about how we can optimally exploit specific NK cell subsets with specialized functions to orchestrate efficacious immune responses against cancer are expected to give better play to the antitumor effect of NK cells.
